# Clinical and Molecular Validation of the Very Favorable IMDC Risk Group in Metastatic Renal Cell Carcinoma

**DOI:** 10.1001/jamanetworkopen.2026.7030

**Published:** 2026-04-15

**Authors:** Martin Zarba, Eddy Saad, Karl Semaan, Talal El Zarif, Evan Ferrier, Connor Wells, Razane El Hajj Chehade, Naveen S. Basappa, Hedyeh Ebrahimi, Mahrukh Huseni, Romain Banchereau, Rana R. McKay, Lori Wood, Benoit Beuselinck, Cristina Suárez, Kosuke Takemura, Aly-Khan A. Lalani, Haoran Li, Lavinia Anne Spain, Arnoud J. Templeton, Thomas B. Powles, Georg A. Bjarnason, Guillermo de Velasco, Toni K. Choueiri, Daniel Y. C. Heng

**Affiliations:** 1 Department of Medical Oncology, Arthur JE Child Comprehensive Cancer Centre, Calgary, Alberta, Canada; 2 Department of Medical Oncology, Dana-Farber Cancer Institute, Harvard Medical School, Boston, Massachusetts; 3 Yale University, New Haven, Connecticut; 4 University of Calgary, Calgary, Alberta, Canada; 5 Department of Medical Oncology, Cross Cancer Institute, University of Alberta, Edmonton, Alberta, Canada; 6 Department of Medical Oncology, City of Hope Comprehensive Cancer Center, Duarte, California; 7 Genentech, San Francisco, California; 8 Department of Medical Oncology, University of California San Diego Health, La Jolla; 9 Division of Medical Oncology, Queen Elizabeth II Health Sciences Centre, Dalhousie University, Halifax, Nova Scotia, Canada; 10 Department of Medical Oncology, Universitair Ziekenhuis Leuven, Leuven, Belgium; 11 Medical Oncology, Vall d’Hebron Institute of Oncology, Vall d’Hebron University Hospital, Barcelona, Spain; 12 Cancer Institute Hospital, Japanese Foundation for Cancer Research, Tokyo, Japan; 13 Department of Medical Oncology, McMaster University, Hamilton, Ontario, Canada; 14 Department of Medical Oncology, Huntsman Cancer Institute, University of Utah, Salt Lake City; 15 Department of Medical Oncology, Peter MacCallum Cancer Centre, Melbourne, Victoria, Australia; 16 Department of Medical Oncology, St Claraspital, Basel, Switzerland; 17 Department of Medical Oncology, Barts Cancer Institute, Queen Mary University of London, London, United Kingdom; 18 Department of Medical Oncology, Sunnybrook Odette Cancer Centre, Toronto, Ontario, Canada; 19 Medical Oncology Department, Hospital Universitario 12 de Octubre, Madrid, Spain

## Abstract

**Importance:**

The International Metastatic Renal Cell Carcinoma Database Consortium (IMDC) risk criteria stratify metastatic renal cell carcinoma (mRCC) into favorable, intermediate, and poor risk groups, but heterogeneity within the favorable risk category remains poorly understood.

**Objective:**

To evaluate a proposed very favorable subgroup (tier 1: Karnofsky Performance Status ≥90%; diagnosis to treatment ≥3 years; and no brain, liver, and bone metastases) and characterize its molecular and clinical features.

**Design, Setting, and Participants:**

This retrospective cohort study analyzed IMDC data, from January 2015 to September 2024, of patients with favorable risk mRCC (tier 1 and tier 2 [favorable and not tier 1]). Molecular profiling leveraged IMmotion151 (A Study of Atezolizumab in Combination With Bevacizumab Versus Sunitinib in Participants With Untreated Advanced Renal Cell Carcinoma) trial data with whole-exome sequencing, RNA sequencing, and programmed cell death ligand 1 immunohistochemistry.

**Exposures:**

Systemic standard of care treatments for mRCC, which include vascular endothelial growth factor receptor targeted therapy (VEGF-TT [sunitinib or pazopanib]), immune-oncology–VEGF (IO-VE [pembrolizumab and axitinib, pembrolizumab and lenvatinib, nivolumab and cabozantinib, or avelumab and axitinib]), and 2 IO (IO-IO [ipilimumab and nivolumab]) regimens.

**Main Outcomes and Measures:**

The primary end point of this study was overall survival (OS) at 2 years of the favorable risk group and in the tier 1 and tier 2 subgroups with the different treatment options. Secondary end points included time to next treatment, treatment duration, and overall response rate. Outcomes were compared across treatment types: VEGF-TT, IO-VE, and IO-IO.

**Results:**

Among 641 patients with favorable risk mRCC (median [IQR] age, 65 [58-71] years; 475 males [74.1%]), 176 (27.5%) were in tier 1, and 465 (72.5%) were in tier 2. Those in tier 1 met criteria for a very favorable subgroup, characterized by similar age and treatment distribution but lower rates of sarcomatoid features; more patients with only 1 metastatic site; and an absence of brain, bone, and liver metastases compared with patients in tier 2 with favorable risk. Patients in tier 1 showed a median OS of 79.1 (95% CI, 73.7 to not reached) months vs 54.5 (95% CI 45.5-67.7) months in tier 2 (*P* < .001) and distinct molecular features: high polybromo-1 alterations (64.7%), low *BRCA1*-associated protein 1 alterations (8.8%), and programmed cell death ligand 1 positivity (21.9%); transcriptomics revealed less immune-infiltrated tumors (8.5% immunogenic clusters) than in the other subgroups. Clinically, IO-IO underperformed in tier 1, with a 2-year OS of 73.1% (95% CI, 49.1%-97.1%) vs 89.1% (95% CI, 79.0%-99.2%) with IO-VE and 92.4% (95% CI, 86.5%-98.3%) for VEGF-TT (IO-IO hazard ratio, 3.64 [95% CI, 1.49-9.06]; *P* = .005), and a lower overall response rate (26.3% vs 63.3% in IO-VE and 57.0% in VEGF-TT).

**Conclusions and Relevance:**

In this cohort study, the very favorable risk subgroup had a less immunogenic molecular profile and superior outcomes from VEGF-containing regimens (VEGF-TT and IO-VE) compared with the favorable risk group. The IO-IO combination showed significantly worse survival in this population, suggesting that VEGF inhibition remains essential for optimal outcomes.

## Key Points

**Question:**

Can the International Metastatic Renal Cell Carcinoma Database Consortium (IMDC) favorable risk group in metastatic renal cell carcinoma be redefined to identify a biologically and clinically distinct very favorable subgroup with different treatment outcomes?

**Findings:**

In this cohort study of 641 patients from the IMDC, a very favorable risk subgroup defined by clinical criteria was found to have significantly longer overall survival and distinct treatment benefit compared with patients in the favorable risk group. Molecular validation using tumors from the IMmotion151 trial showed that the very favorable subgroup was enriched for angiogenic features and less immunogenic profiles, with improved outcomes on vascular endothelial growth factor receptor–based therapies and inferior outcomes with 2 immune-oncology regimens.

**Meaning:**

The findings of this study suggest that refining the IMDC favorable risk category identified a biologically distinct subgroup that may benefit from tailored treatment strategies and may inform future clinical trial design.

## Teaser

This cohort study evaluates whether the International Metastatic Renal Cell Carcinoma Database Consortium (IMDC) risk criteria that stratify metastatic renal cell carcinoma (mRCC) can be redefined to identify a biologically and clinically distinct very favorable subgroup with different treatment outcomes.

## Introduction

The treatment of metastatic renal cell carcinoma (mRCC) has evolved substantially in recent years with the introduction of combination therapies. These include a combination of 2 immune-oncology (IO) agents, such as ipilimumab and nivolumab (IO-IO), as well as combinations of vascular endothelial growth factor receptor targeted therapies (VEGF-TTs) and IO–VEGF agents (IO-VE).^1,2^ These advancements have expanded first-line therapeutic options and altered the treatment landscape for patients with mRCC.

The prognostic model developed by the International Metastatic Renal Cell Carcinoma Database Consortium (IMDC) in 2009 has been instrumental for patient stratification in phase 3 clinical trials, which established the efficacy of these contemporary first-line combination therapies. Originally validated in patients receiving VEGF-TT, the IMDC model incorporates 6 prognostic factors: (1) time from initial diagnosis to systemic therapy less than 1 year; (2) Karnofsky Performance Status (KPS) less than 80%, in which scores range from 0 to 100, with higher percentages indicating better functioning; (3) low serum hemoglobin; (4) elevated platelet count; (5) elevated absolute neutrophil count; and (6) corrected serum calcium above the upper limit of normal. Based on these criteria, patients are classified into 3 risk groups: favorable (0 risk factors), intermediate (1 or 2 risk factors), and poor (≥3 risk factors).^3^ This model continues to be relevant in the modern era with IO treatment, as it remains prognostic across risk groups and continues to provide valuable prognostic information and guides treatment decisions, assisting in predicting the effectiveness of systemic therapies.

Despite its clinical utility, the role of immunotherapy combinations in patients with favorable risk mRCC, as classified by the IMDC model, remains a topic of debate. Although not powered to do so, to our knowledge, IO combinations’ pivotal trials were not able to demonstrate any benefit in overall survival (OS) in this subgroup.^2,4,5,6,7^ Evidence suggests that this group is characterized by an angiogenic rather than an immunogenic profile,^8^ which could explain this lack of substantial benefit with IO-containing regimens.

Moreover, a subgroup of patients within the favorable risk group with exceptionally good prognosis was initially described by Schmidt et al^9^ in 2021. This subgroup was called very favorable and was defined as a subgroup within the favorable risk group that also had a time from primary diagnosis to systemic therapy of 3 or more years; a KPS of 90% or 100%; and an absence of brain, liver, or bone metastasis.^9^ In the Schmidt et al study,^9^ patients with all of these variables had a median OS of 64.8 (95% CI, 58.8-70.8) months compared with 45.6 (95% CI, 42.0-50.4) months in the entire favorable cohort (hazard ratio [HR], 1.84 [95% CI, 1.56-2.20]; *P* < .001). This very favorable subgroup presents a challenge in the current era of IO combinations, as it is not represented as such in clinical trials; and as a result, there is a lack of proper evidence to guide clinical decisions, which warrants further investigation to clarify the optimal therapeutic approach for this population.

## Methods

### Study Population

This retrospective cohort study was conducted using the IMDC to identify patients with mRCC who were classified as having favorable risk, irrespective of histology, and who initiated first-line systemic therapy between January 2015 and September 2024. Data were collected from hospital and pharmacy records using standardized database collection templates. Institutional review board approval was obtained from each participating center. Waivers of consent were approved by the local research ethics boards of all participating institutions to facilitate maximal capture of the local patients populations and minimize bias; all patient data were deidentifed. This study was conducted and reported in accordance with the Strengthening the Reporting of Observational Studies in Epidemiology (STROBE) reporting guideline for cohort studies.

This study included only contemporary, guideline-recommended treatment regimens reflective of clinical practice. IO-VE combinations were defined as follows: axitinib and pembrolizumab, cabozantinib and nivolumab, lenvatinib and pembrolizumab, or axitinib and avelumab. VEGF-TT was defined as sunitinib or pazopanib. IO-IO was defined as ipilimumab and nivolumab. Patients were excluded if they received any first-line treatment outside of these regimens or if IMDC risk classification could not be determined due to missing data.

Patients included in the study met the IMDC favorable risk classification criteria, defined as the absence of any of the IMDC prognostic factors described previously. Within the favorable-risk cohort, we defined 2 subgroups: tier 1 (very favorable risk), including patients with a KPS of 90% or more; the time from diagnosis to systemic therapy of 3 or more years; and the absence of brain, liver, or bone metastases, and tier 2 (standard favorable risk), including patients who met IMDC favorable risk criteria but did not fulfill all tier 1 criteria.

To validate the prognostic implications of these subgroups, OS analysis was conducted using the Kaplan-Meier method, with statistical significance determined by a log-rank test (*P* < .05), including an overall comparison of tier 1, tier 2, intermediate, and poor risk groups. A separate pairwise survival analysis was performed to compare tier 1 and tier 2 subgroups directly.

### Molecular Characterization of the Very Favorable Risk Group in the IMmotion151 Clinical Trial

In an effort to uncover the genomic and transcriptomic underpinnings of the very favorable risk group, we leveraged data from the IMmotion151 (A Study of Atezolizumab in Combination With Bevacizumab Versus Sunitinib in Participants With Untreated Advanced Renal Cell Carcinoma) phase 3 trial. In this trial, patients with previously untreated advanced RCC with clear cell component or sarcomatoid histology were randomized to receive either atezolizumab and bevacizumab or sunitinib.^10^ Patients belonging to the very favorable risk group were identified using the 3 previously described criteria. Baseline characteristics were compared among the 4 risk groups, as well as between the very favorable and standard favorable groups, using the Fisher test for categorical variables and the Kruskal-Wallis rank sum test for continuous variables. In cases of contingency tables larger than 2 × 2, the Monte Carlo simulation was used to compute *P* values. No additional covariates were included in the transcriptomic analyses, as data on key variables such as body mass index, smoking status, and genetic ancestry were unavailable, and age and sex were not incorporated given the lack of consistent evidence for biologically meaningful differences in advanced RCC.

### PD-L1 IHC and Somatic Variant Profiling

Programmed cell death ligand 1 (PD-L1) immunohistochemistry (IHC) was performed using the SP142 assay (Ventana). Profiling of somatic variants was performed using the Foundation One T7 assay (Foundation Medicine Inc).^11^ Briefly, somatic variants and copy number alterations were detected in 395 genes. Somatic variants were characterized as short variant, loss, rearrangement, truncation, amplification, deletion, duplication, or fusion. Genomic alterations in each risk group were visualized using the ComplexHeatmap package in R, version 2.16.0 (R Project for Statistical Computing).^12^ The frequency of alterations in each gene was compared between risk groups using the Fisher test.

### Transcriptomic Analysis

RNA sequencing data from the IMmotion151 cohort was processed as described previously.^10^ Samples with fewer than 15 000 genes detected (indicating low library complexity) were excluded. Genes not expressed in any of the samples were filtered out. For subsequent analysis, log-transformed transcripts per million (log2[transcripts per million + 1]) counts were used. Single sample gene set enrichment analysis (ssGSEA) was computed using the GSVA package in R to obtain sample-level GSEA scores for the Reactome Pathway Database and the Pathway Interaction Database.^13^ Differential pathway activity was assessed using the nonparametric Kruskal-Wallis test on ssGSEA scores with Benjamini-Hochberg false discovery rate correction (Cochran *Q* test [*P* < .05 considered significant). For pathways that passed the false discovery rate threshold, a pairwise Mann-Whitney test was performed to assess differences between individual risk groups. Samples were further classified into 1 of 7 previously described molecular clusters using a random forest machine learning algorithm.^10^ The proportion of different clusters was assessed between risk groups using the Fisher test.

### Outcomes Analysis on the IMDC Cohort

The primary end point of this study was OS at 2 years of the favorable risk group and in the tier 1 and tier 2 subgroups with the different treatment options. Secondary end points included time to next treatment (TTNT), treatment duration (TD), and overall response rate (ORR). OS was defined as the time from initiation of first-line systemic therapy to death from any cause, with censoring at the last follow-up. TTNT was defined as the time from first-line therapy initiation to the initiation of second-line therapy or death, with censoring at the last follow-up. TD was defined as the duration of first-line systemic therapy until discontinuation for any reason, with censoring at the last follow-up. TTNT and TD were chosen over progression-free survival or time to treatment failure due to the potential for sustained immunotherapy effects beyond treatment discontinuation and the possibility of treatment beyond first progression.

Response to treatment was assessed based on physician assessment using Response Evaluation Criteria in Solid Tumors (RECIST), version 1.1 principles.^14^ ORR was calculated as the proportion of patients achieving either complete response or partial response as their best response to first-line systemic therapy, according to local investigators.

### Statistical Analysis

Comparisons of patient outcomes were performed across IMDC favorable risk subgroups within each treatment cohort. OS, TTNT, and TD were estimated using the Kaplan-Meier method, with significance assessed by a log-rank test (*P* < .05). Baseline demographic and clinical characteristics were summarized using proportions for categorical variables and medians with IQRs for continuous variables. χ^2^ Tests were used to assess differences in baseline characteristics between risk groups in the IO-IO and IO-VE cohorts compared with the VEGF-TT cohort, with statistical significance set at a 2-sided *P* < .05. Analyses were conducted using a complete-case approach, whereby only patients with available data for the variables of interest were included in each analysis, and no imputation was performed for missing values. The proportional hazards assumption was formally assessed using Schoenfeld residuals, with correlation between scaled residuals and time evaluated to identify potential time-dependent effects. All statistical analyses were performed using SAS, version 9.4 (SAS Institute Inc).

## Results

### Baseline Characteristics

A total of 641 patients with favorable risk mRCC were included (median [IQR] age, 65 [58-71] years; 166 females [25.9%] and 475 males [74.1%]). Of these, 176 (27.5%) met the criteria for the very favorable risk subgroup (tier 1), and 465 (72.5%) were classified as tier 2 favorable risk. Most patients had undergone prior nephrectomy (624 of 642 patients [97.2%]); tier 1 patients exhibited fewer patients with more than 1 metastatic site and lower rates of sarcomatoid features compared with tier 2. Baseline characteristics are summarized in the Table.

**Table.** Baseline Characteristics of Study Participants

Characteristic	Patients, No. (%)		
	Favorable risk (N = 641)	Tier 1 favorable or very favorable risk (n = 176)	Tier 2 favorable risk (n = 465)
Age, median (IQR), y	65 (58-71)	66 (59-71)	65 (58-71)
Sex			
Female	166 (25.9)	53 (30.1)	113 (24.3)
Male	475 (74.1)	123 (69.9)	352 (75.7)
Sarcomatoid features^a^	24/466 (5.2)	2/140 (1.4)	22/326 (6.8)
Nonclear cell histology^a^	70/552 (12.7)	18/161 (11.2)	52/391 (13.3)
Nephrectomy^a^	624/642 (97.2)	174 (98.9)	450 (96.6)
>1 Metastatic site^a^	453/595 (77.0)	116/166 (69.9)	342/429 (79.7)
Site of metastasis^a^			
Lung	417/633 (65.9)	108 (61.0)	309/456 (67.8)
Brain	35/617 (5.7)	0	35/440 (8.0)
Bone	130/625 (20.8)	0	130/448 (29.0)
Liver	88/620 (14.2)	0	88/443 (19.9)
First-line treatment			
IO-IO	97 (15.1)	22 (12.5)	75 (16.1)
IO-VE	183 (28.5)	59 (33.5)	124 (26.7)
Pembrolizumab and axitinib	102 (15.9)	34 (19.3)	68 (14.6)
Avelumab and axitinib	22 (3.4)	6 (3.4)	16 (3.4)
Nivolumab and cabozantinib	32 (5.0)	11 (6.3)	21 (4.5)
Pembrolizumab and lenvatinib	27 (4.2)	8 (4.5)	19 (4.1)
VEGF-TT	361 (56.3)	95 (54.0)	266 (57.2)

Abbreviations: IO-IO, 2 immune oncology; IO-VE, IO–vascular endothelial growth factor receptor; VEGF-TT, vascular endothelial growth factor receptor targeted therapy.

^a^ Denominators may differ because patients with missing data are not included.

### Clinical Validation

**Figure 1.**
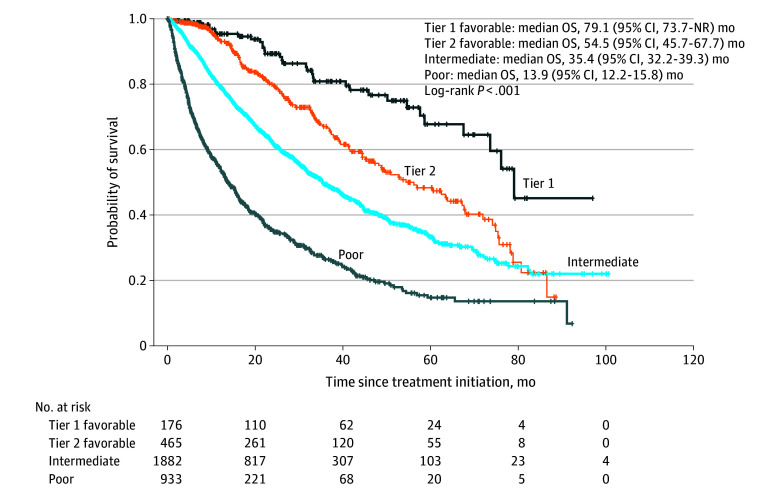
Survival Plot of Overall Survival (OS) of the Entire International Metastatic Renal Cell Carcinoma Database Consortium (IMDC) of Tier 1 and 2 Favorable, Intermediate, and Poor Risk Groups Survival analysis according to the IMDC Risk Classification. Vertical marks indicate censoring; NR, not reached.

Survival analysis across the entire IMDC cohort demonstrated a significant difference in OS among poor risk, intermediate risk, and tier 2 and tier 1 favorable risk groups, regardless of first-line treatment. The median OS for the poor risk group was 13.9 (95% CI, 12.2-15.8) months, 35.4 (95% CI, 32.2-39.3) months for the intermediate risk group, 54.5 (95% CI, 45.5-67.7) months for the tier 2 favorable group, and 79.1 (95% CI, 73.7 to not reached) months for the tier 1 (very favorable) risk subgroup (log-rank *P* < .001) (Figure 1). When comparing only tier 1 and tier 2, irrespective of treatment, the survival difference maintained its significance (79.1 months vs 54.5 months; *P* = .001).

### Molecular Validation

**Figure 2.**
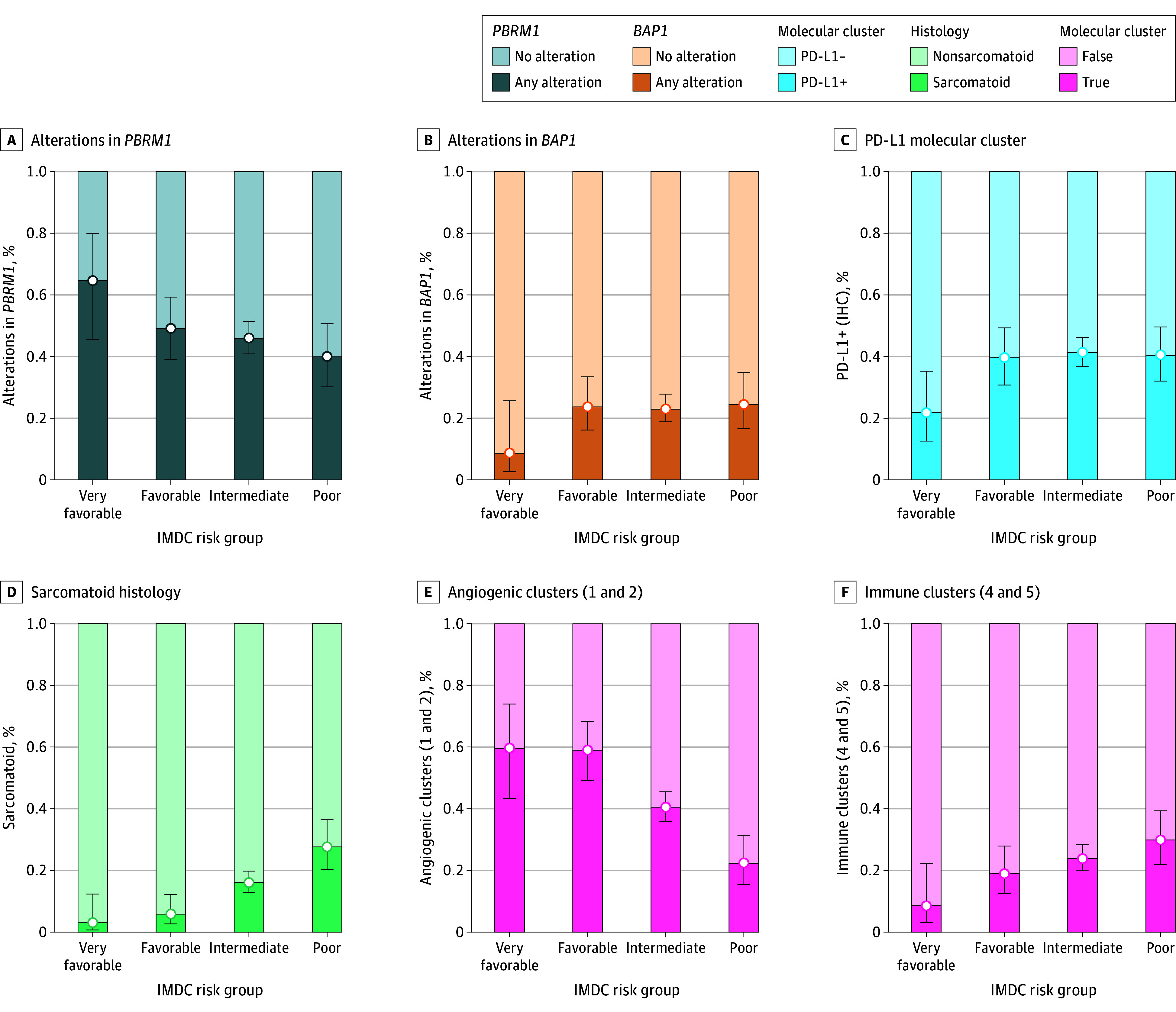
Bar Graphs of Genomic and Histological Characterization of the Very Favorable Risk Group in the Cohort of A Study of Atezolizumab in Combination With Bevacizumab Versus Sunitinib in Participants With Untreated Advanced Renal Cell Carcinoma (IMmotion151) Trial Comparisons of alteration frequencies and proportions of angiogenic and immune molecular clusters between the very favorable group and all other risk groups. *P* values were calculated with the Fisher exact test. *BAP1* indicates *BRCA1*-associated protein 1; IHC, immunohistochemistry; IMDC, International Metastatic Renal Cell Carcinoma Database Consortium; *PBRM1*, polybromo-1; PD-L1, programmed cell death ligand 1, +, 1% or more of tumor-infiltrating immune cells expressing PD-L1; −, less than 1% tumor-infiltrating immune cells expressing PD-L1.

We sought to explore the biological characteristics of tumors across the IMDC risk groups, including the very favorable group, using data from patients enrolled in the IMmotion151 trial. Among 913 included patients, 64 (7%) were classified in the very favorable category. We comprehensively characterized the somatic variants of 715 tumors stratified by IMDC risk groups, including 34 from patients in the very favorable group. Among the top 15 mutated genes in our cohort (eFigure 1 in Supplement 1), we identified somatic variants in canonical clear cell RCC genes such as Von Hippel–Lindau (*VHL*), SET domain containing 2 (*SETD2*), *BRCA1*-associated protein 1 (*BAP1*), tumor protein 53 (*TP53*), cyclin-dependent kinase inhibitor 2A/B (*CDKN2A/B*), lysine-specific demethylase 5C (*KDM5C*), phosphatase and tensin homolog (*PTEN*), and mechanistic target of rapamycin kinase (*MTOR*). However, it was notable that the frequency of these variants was variable across the IMDC risk groups. For instance, polybromo-1 (*PBRM1*) variants were not significantly higher in the very favorable risk group (22 [64.7%]) compared with the favorable group (58 [49.2%]) (*P* = .12) and were significantly higher than in the intermediate (208 [46.0%]) (*P* = .049) and poor risk (44 [40.0%]) (*P* = .02) groups. An opposite trend that occurred least commonly in the very favorable risk group was observed in *BAP1* (3 [8.8%]) and *CDKN2A/B* (3 [8.8%]) genomic alterations and most commonly in the poor risk group (*BAP1*: 27 [24.5%] and *CDKN2A/B*: 33 [30.0%]) (*P* = .055 [*BAP1*] and *P* = .01 [*CDKN2A/B*]) (Figure 2).

Patients with very favorable risk showed the lowest rates of PD-L1 positivity on IHC (14 [21.9%]), significantly lower than those in the favorable (54 [39.7%]) (*P* = .02), intermediate (232 [41.4%]) (*P* = .003), and poor (62 [40.5%]) (*P* = .01) risk groups (Figure 2). Furthermore, the rate of tumors with sarcomatoid features, associated with more aggressive disease, increased steadily from 2 (3.1%) in the very favorable risk group to 8 (5.9%) in the favorable (*P* = .51), 90 (16.1%) in the intermediate (*P* = .003), and 42 (27.6%) in the poor (*P* < .001) risk groups (Figure 2).

Using RNA sequencing data derived from 821 tumors, the distribution of the previously described IMmotion151 molecular clusters was examined. The proportion of tumors falling in the angiogenic clusters (1 and 2)^8^ was higher in the very favorable (59.6%) and favorable (59.1%) risk groups (*P* > .99) compared with the intermediate (40.5%) (*P* = .01) and poor (22.4%) (*P* < .001) risk groups. In contrast, the rate of the immunogenic clusters (4 and 5) progressively increased from 8.5% in the very favorable to 18.9% in the favorable (*P* = .11), 23.8% in the intermediate (*P* = .02), and 29.9% in the poor (*P* = .003) IMDC risk groups (Figure 2 and eFigure 2 in Supplement 1). These findings were corroborated by ssGSEA (eTable in Supplement 2); tumors from patients with very favorable risk were associated with enriched VEGF ligand-receptor interaction pathways when compared with patients with favorable risk or those with intermediate and poor risk combined (eFigure 2 in Supplement 1), while associated with a lower enrichment for adaptive and innate immunity pathways when compared with the intermediate and poor risk combined (eFigure 2 in Supplement 1). Taken together, these findings point toward a more angiogenic and less immunogenic phenotype observed in the very favorable risk group.

### Clinical Outcomes of the IMDC Cohort

**Figure 3.**
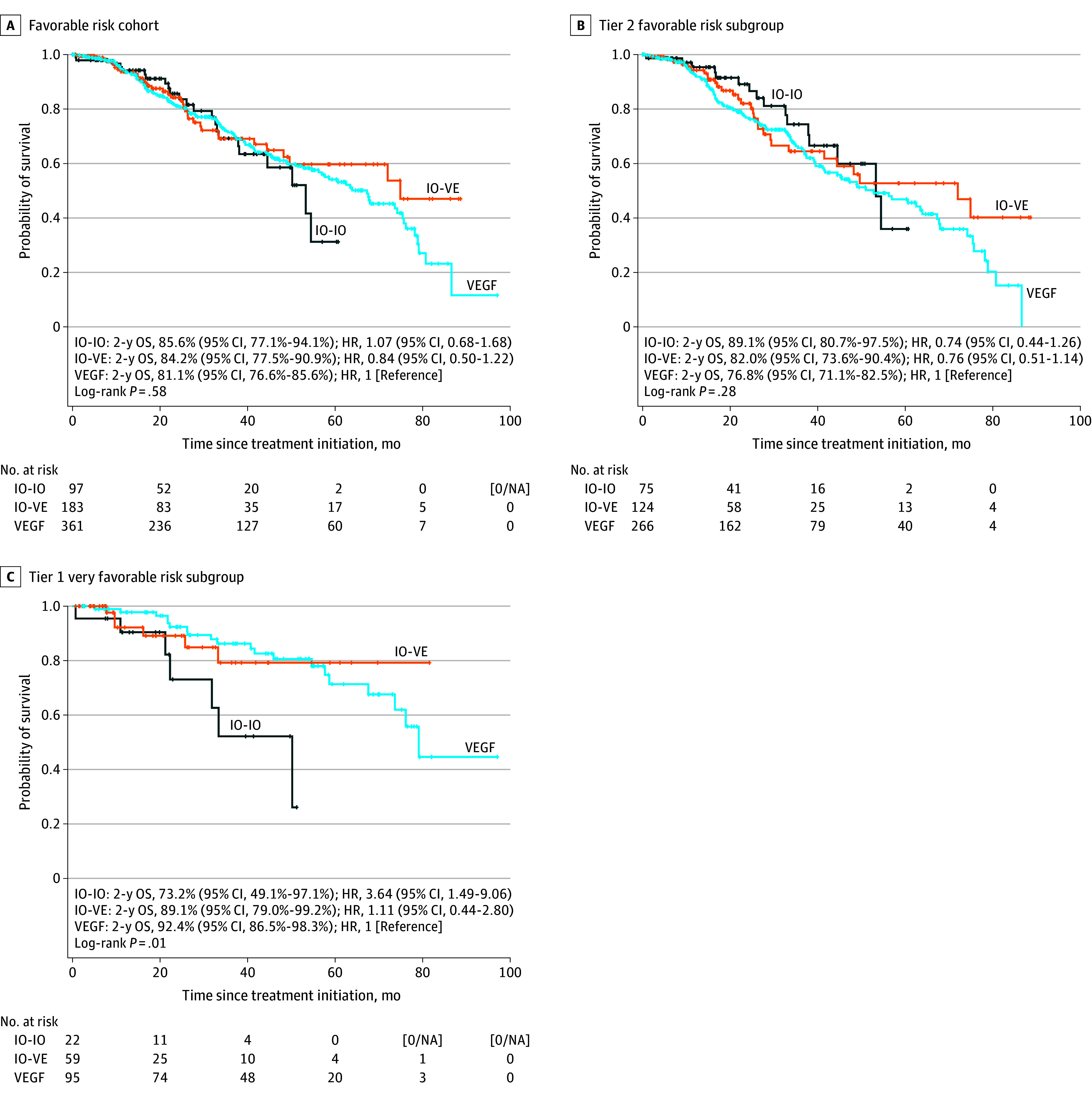
Survival Plots of Kaplan-Meier Curves for Overall Survival (OS) Vertical marks indicate censoring; HR, hazard ratio; IO-IO, 2 immune oncology; IO-VE, IO–vascular endothelial growth factor receptor; VEGF, vascular endothelial growth factor receptor.

With a median follow-up of 32.9 (95% CI, 29.7-36.3) months, we did not find a statistically significant difference in the primary end point of 2-year OS between treatment groups in the overall favorable risk cohort or within the tier 2 favorable subgroup. In the favorable risk cohort, the estimated 2-year OS rates were 81.1% (95% CI, 76.6%-85.6%) for patients treated with VEGF-TT, 84.2% (95% CI, 77.5%-90.9%) for those receiving an IO-VE combination (HR, 0.84 [95% CI, 0.50-1.22]; *P* = .36), and 85.6% (95% CI, 77.1%-94.1%) for those treated with IO-IO (HR, 1.07 [95% CI, 0.68-1.68]; *P* = .76) (Figure 3A). In the tier 2 favorable subgroup, the 2-year OS rate was 76.8% (95% CI, 71.1%-82.5%) for VEGF-TT, 82.0% (95% CI, 73.6%-90.4%) for the IO-VE combination (HR, 0.76 [95% CI, 0.51-1.14]; *P* = .19), and 89.1% (95% CI, 80.7%-97.5%) for IO-IO (HR, 0.74 [95% CI, 0.44-1.26]; *P* = .27) (Figure 3B).

In contrast, within the very favorable risk subgroup (tier 1), a lower 2-year OS rate was observed among patients receiving IO-IO compared with IO-VE or VEGF-TT therapies. In this group, the estimated 2-year OS rate was 92.4% (95% CI, 86.5%-98.3%) for VEGF-TT, 89.1% (95% CI, 79.0%-99.2%) for the IO-VE combination, and 73.1% (95% CI 49.1%-97.1%) for IO-IO (*P* = .01). Using VEGF-TT as the reference, the HR for IO-VE was 1.11 (95% CI, 0.44-2.80; *P* = .83), and the HR for IO-IO was 3.64 (95% CI, 1.49-9.06; *P* = .005) (Figure 3C). When the analysis was restricted to patients with clear cell histology, this difference was maintained, with a statistically significant poorer OS in the IO-IO group (*P* = .007).

**Figure 4.**
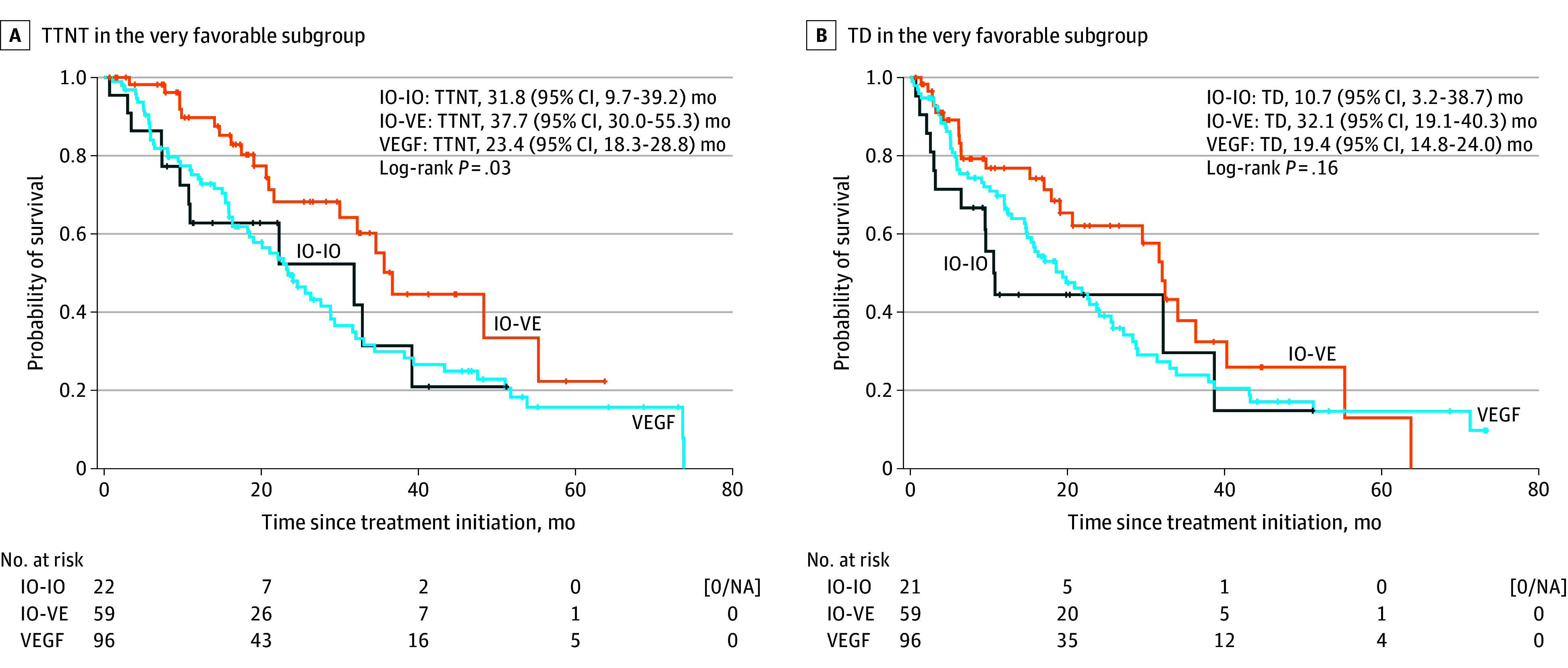
Survival Plots of Time to Next Treatment (TTNT) and Treatment Duration (TD) in the Very Favorable Subgroups Vertical marks indicate censoring; IO-IO, 2 immune oncology; IO-VE, IO–vascular endothelial growth factor receptor; VEGF, vascular endothelial growth factor receptor.

No evidence of violation of the proportional hazards assumption was observed, as Schoenfeld residuals showed no significant correlation with time for tier 1 (*r *= 0.04; *P* = .83), tier 2 (*r* = 0.12; *P* = .16), or the favorable risk group (*r* = 0.11; *P* = .14). In the tier 1 subgroup, the TTNT was significantly longer with IO-VE combinations over IO-IO or VEGF-TT (log-rank *P* = .03), with a median of 23.4 (95% CI, 18.3-28.8) months for VEGF-TT, 37.7 (95% CI, 30.0-55.3) months for the IO-VE combination, and 31.8 (95% CI, 9.7-39.2) months for IO-IO (Figure 4A). TD did not significantly favor the IO-VE combination (log-rank *P* = .16), with a median duration of 32.1 (95% CI, 19.1-40.3) months compared with 10.7 (95% CI, 3.2-38.7) months for IO-IO and 19.4 (95% CI, 14.8-24.0) months for VEGF-TT (Figure 4B). When evaluating response rates within the very favorable subgroup, the ORR was significantly lower for IO-IO (*P* = .02), with response rates of 26.3% for this regimen compared with 63.3% for the IO-VE combination and 57.0% for VEGF-TT.

Additional secondary outcomes including TTNT, TD, and ORR differed significantly by treatment strategy. In the overall favorable risk cohort, IO-VE therapy was associated with longer TTNT (median 32.2 [95% CI, 22.7-42.6] months) compared with IO-IO (median 22.3 [95% CI, 15.3-35.3] months) and VEGF-TT (median 15.4 [95% CI, 13.8-17.3] months) and TD (median 22.2 [95% CI, 17.9-29.5] months) compared with IO-IO (median 7.6 [95% CI, 4.8-14.3] months) and VEGF-TT (median 12.0 [95% CI, 10.3-13.3] months) (*P* < .001 in both outcomes), along with a higher ORR for IO-VE (54.4%) vs for IO-IO (37.9%) and for VEGF-TT (38.7%) (*P* = .002). Similar findings were observed in the tier 2 favorable subgroup, in which IO-VE demonstrated superior TTNT (median 26.4 [95% CI, 22.4-39.1] months; *P* < .001), TD (median 21.2 [95% CI, 15.9-22.7] months; *P* < .001), and ORR (50.5%; *P* = .003) compared with IO-IO and VEGF-TT. In contrast, within the tier 1 subgroup, IO-VE was associated with longer TTNT (median 37.7 [95% CI, 30.0-55.3] months) and higher ORR (63.3%) compared with IO-IO (TTNT, median 31.8 [95% CI, 9.7-39.2] months; ORR, 26.3%) and with VEGF-TT (TTNT, median 23.4 [95% CI, 18.3-28.8] months; ORR, 57.0%) with *P* = .02 for TTNT, and *P* = .02 for ORR, although there were no statistically significant differences in TD.

A look at causes of death in the very favorable subgroup treated with IO-IO revealed that most patients died due to disease progression. Among the 5 documented deaths that we have information about, 4 were associated with progressive disease, while 1 patient death was associated with immune-related adverse events, including myocarditis, hepatitis, and myasthenia gravis.

## Discussion

This cohort study refines the prognostic stratification of patients with mRCC classified as favorable risk by the IMDC criteria. By subdividing this group into tier 1 (very favorable) and tier 2 (favorable) subgroups, we were able to demonstrate clinically meaningful and statistically significant differences in survival irrespective of treatment, as well as differences in treatment efficacy. This refined classification may serve as a crucial tool for optimizing patient selection for specific therapeutic strategies, ensuring that treatments are better tailored to individual risk profiles.

The rapid advancements in genetic and molecular classifications of mRCC are revolutionizing our understanding of the disease. These efforts are invaluable in deciphering the underlying biology and identifying predictive factors of response to different treatment strategies. However, despite these advances, clinical criteria remain the primary determinants of prognosis and treatment selection in clinical practice, which raises the question of whether clinical and biochemical patterns can act as a surrogate of molecular features. Our study reinforces the importance of clinical variables in predicting outcomes and introduces an additional layer of refinement that could enhance decision-making in clinical settings. By establishing that the tier 1 (very favorable) and tier 2 (favorable) subgroups show different survival outcomes and treatment responses, our findings provide a framework for further refining risk stratification in mRCC.

One of the key findings of our study is that the very favorable risk subgroup not only demonstrated significantly better OS than the tier 2 favorable subgroup but also responded differently to first-line therapies. Importantly, our analysis highlights that VEGF-TT, either as monotherapy or in combination with immune checkpoint inhibitors (IO-VE), remains a crucial component of treatment for patients in the very favorable subgroup. The data suggest that treatment strategies that include a VEGF-TT component are essential for this population, as their omission may lead to suboptimal outcomes. Notably, our findings indicate that patients treated with IO-IO within the very favorable risk group experienced significantly worse outcomes, with a higher incidence of early mortality despite their inherently favorable prognosis. This observation aligns with prior reports suggesting that angiogenesis-driven tumors may not derive the same benefit from IO-IO therapy as those with a more inflammatory tumor microenvironment.^8^

Our molecular analysis further supports a biological rationale for the superior outcomes seen with VEGF-TT in the very favorable risk subgroup. Tumors in this very favorable group were substantially enriched for angiogenesis-related molecular clusters (clusters 1 and 2 in IMmotion151), which have been previously associated with improved responses to VEGF inhibition. Moreover, this subgroup demonstrated lower PD-L1 expression and reduced prevalence of immunogenic clusters, features suggestive of a microenvironment less responsive to immune checkpoint blockade alone. These findings suggest that the efficacy of VEGF-TT in patients with very favorable risk may stem from an underlying angiogenesis-driven tumor biology, reinforcing the importance of VEGF inhibition as a central component of first-line therapy for this population. However, the molecular validation of the very favorable subgroup is limited by a small sample size, which reduces statistical power and yields imprecise effect estimates; therefore, these findings should be interpreted as exploratory and hypothesis-generating and require confirmation in independent cohorts enriched for patients with very favorable risk.

It is possible that patients in the very favorable subgroup derive a substantial portion of their therapeutic benefit through inhibition of angiogenesis. Support for this hypothesis can be found in translational studies from the IMmotion151 trial, in which angiogenesis-enriched molecular clusters (clusters 1 and 2) were associated with improved outcomes with VEGF-TT.^15^ Moreover, patients with pancreatic metastases, another hallmark of indolent, angiogenesis-dependent disease, also appear to respond favorably to VEGF-TT.^14^ These findings suggest a potential biological rationale for the greater efficacy of VEGF-containing regimens in this population and reinforce the need for further investigation into biomarkers that can better predict treatment benefits.

Despite the lack of OS benefit in the favorable risk group with IO-IO in the CheckMate 214 trial^2^ and a subsequent lack of approval of this combination in some jurisdictions, we observed that this regimen was still prescribed occasionally in our cohort, although it was the least-selected option. This treatment showed no survival differences in the tier 2 subgroup, but it was significantly different in tier 1. In the very favorable risk subgroup, in which only 22 patients received IO-IO, survival was significantly worse, showing a higher incidence of early deaths. The predominant cause of death in this cohort was disease progression, underscoring the importance of including VEGF inhibition in treatment regimens for these patients. Rather than concluding that ipilimumab and nivolumab should be categorically avoided, our findings suggest that the absence of a VEGF-TT component may be detrimental to patients in the very favorable subgroup. Additionally, 1 patient with immune-related toxic effects leading to death was reported. These findings raise important concerns regarding treatment selection and highlight the potential risks associated with administering IO-IO therapy in patients with a very favorable prognosis. While the use of ipilimumab and nivolumab in patients in the IMDC favorable risk group remains debated, long-term data, including the 99.1-month follow-up, now suggest a trend toward improved OS and higher complete response rates compared with sunitinib,^16^ supporting its rationale in select patients in tier 2; importantly, this approach preserves the option of multiple VEGF-TTs in later lines, unlike the reverse, and aligns with the European Society for Medical Oncology guidelines,^17^ which acknowledge the lack of consensus but do not discourage this strategy.

### Strengths and Limitations

Our study has strengths. It suggests a potential predictive value of this novel classification system, although further validation is warranted. Beyond its role as a prognostic tool, the stratification of patients with favorable risk into tier 1 and tier 2 favorable subgroups provides possible actionable insights for treatment selection. The significantly poorer outcomes observed in patients with very favorable risk treated with IO-IO therapy should give one pause in prescribing this therapy to this subgroup of patients. By integrating this refined classification into clinical practice, we may improve therapeutic decision-making and potentially avoid ineffective treatment strategies for patients with an excellent prognosis.

Although the IMDC is a large dataset, this study has limitations that must be acknowledged. As a retrospective analysis, our study is subject to selection biases and potential confounders that may influence treatment outcomes. As part of our study was based on clinical data, treatment selection was not randomized, and we do not have any insight into the reason why each treatment was chosen (a more aggressive tumor biology or patients with potentially more severe illness with greater tumor volume could have influenced physicians to choose an IO-IO combination). As an example, this study lacks systematic availability of data on race and ethnicity and relevant comorbid conditions (such as body mass index, cardiovascular disease, hypertension, and diabetes), which precluded adjustment for these factors and may have influenced survival outcomes. Also, it is worth mentioning that we included patients with nonclear cell histology to maintain consistency with the original IMDC risk stratification model, but we acknowledge that this inclusion may have influenced the observed outcomes, as nonclear cell RCCs exhibit distinct molecular and clinical characteristics that could impact prognosis and treatment response. Given the evolving treatment landscape in mRCC over the study period, including transitions from VEGF-TT to IO-based combinations, temporal bias may have influenced treatment selection and outcomes, and this factor should be considered in the interpretation of the results.

In addition, only 22 patients in the very favorable subgroup were treated with ipilimumab and nivolumab, which limits the statistical power to draw firm conclusions about the efficacy of this regimen in this specific group. Furthermore, another major limitation is the median follow-up time of our cohort. The CheckMate 214 study^2,16^ required more than 8 years of follow-up to demonstrate a crossover in survival curves among patients with favorable risk, with an observed trend toward better outcomes with IO-IO therapy that was not statistically significant. This raises the question of whether our conclusion, based solely on 2-year OS data, is premature, and whether these results could change with a longer follow-up. Additionally, while we provided evidence supporting the prognostic and predictive utility of this refined classification, prospective validation in randomized clinical trials is essential to confirm our findings. Future studies should aim to validate this classification in independent cohorts and assess its applicability across different treatment landscapes. Prospective validation of the very favorable subgroup could be achieved by incorporating the proposed clinical criteria as a prespecified stratification factor or enrichment criterion in future randomized clinical trials of first-line therapy for mRCC. Patients meeting very favorable criteria could be prospectively identified at trial entry and either analyzed as a dedicated subgroup or enrolled in trials specifically designed to compare VEGF-containing regimens with IO-IO regimens within this population. In addition, prospective registries and biomarker-driven studies could incorporate this classification to assess its reproducibility, prognostic value, and predictive relevance across treatment platforms and disease settings.

## Conclusions

In this cohort study, we provided insights into the prognostic and predictive implications of subclassifying favorable risk mRCC into tier 1 (very favorable) and tier 2 (favorable) subgroups. This study found that the refined classification may enhance prognostication and inform treatment selection, highlighting the critical role of VEGF-TT (alone or in combination) in patients with very favorable risk RCC. The poorer outcomes observed with IO-IO therapy in this subgroup warrant further investigation, and prospective randomized clinical trials will be necessary to refine treatment guidelines. As we continue to integrate molecular and genetic insights into clinical practice, maintaining a focus on robust clinical criteria remains paramount for optimizing outcomes in patients with mRCC.

## Notes

Supplement 1. **eFigure 1.** CoMut Plot Summarizes the Prevalence of Somatic Mutations in the 15 Most Frequently Altered Genes Across IMDC Risk Groups **eFigure 2.** Distribution of the 7 Molecular Clusters in Each Risk Group and ssGSEA Scores for Angiogenic and Immune Pathways Stratified by IMDC Risk Group

Supplement 2. **eTable.** Single Sample Gene Set Enrichment Analysis

Supplement 3.
Data Sharing Statement
