# Supplementary material for: Clinical and Molecular Validation of the Very Favorable IMDC Risk Group in Metastatic Renal Cell Carcinoma
Source: JAMA Netw Open. 2026 Apr 15;9(4):e267030. doi: 10.1001/jamanetworkopen.2026.7030 (PMC13084435; doi:10.1001/jamanetworkopen.2026.7030)
Supplement: Supplement 1. — eFigure 1. CoMut Plot Summarizes the Prevalence of Somatic Mutations in the 15 Most Frequently Altered Genes Across IMDC Risk Groups eFigure 2. Distribution of the 7 Molecular Clusters in Each Risk Group and ssGSEA Scores for Angiogenic and Immune Pathways Stratified by IMDC Risk Group [file jamanetwopen-e267030-s001.pdf]

Supplemental Online Content

Zarba M, Saad E, Semaan K, et al. Clinical and molecular validation of the very favorable imdc risk group in metastatic renal cell carcinoma. *JAMA Netw Open.* 2026;9(4):e267030. doi:10.1001/jamanetworkopen.2026.7030

eFigure 1. CoMut Plot Summarizes the Prevalence of Somatic Mutations in the 15 Most Frequently Altered Genes Across IMDC Risk Groups

eFigure 2. Distribution of the 7 Molecular Clusters in Each Risk Group and ssGSEA Scores for Angiogenic and Immune Pathways Stratified by IMDC Risk Group

This supplemental material has been provided by the authors to give readers additional information about their work.

© 2026 Zarba M et al. *JAMA Network Open*.

eFigure 1. CoMut Plot Summarizes the Prevalence of Somatic Mutations in the 15 Most Frequently Altered Genes Across IMDC Risk Groups

Supplementary fig 1. CoMut plot summarizes the prevalence of somatic alterations in the 15 most frequently altered genes across IMDC risk groups. Each column represents an individual tumor sample, grouped by clinical risk category, while each row corresponds to a specific gene. Distinct colors denote different classes of genomic alterations, including missense mutations, truncating mutations, copy number gains, and copy number losses. The bar plots above the matrix indicate the overall mutational burden per sample, whereas the bar plots on the right summarize the frequency of alterations for each gene within the cohort. This visualization highlights differential genomic alteration patterns across risk groups, including enrichment of angiogenesis-associated genes such as *PBRM1* in lower-risk categories and higher prevalence of adverse prognostic alterations, including *BAP1*, in higher-risk groups, supporting biologically distinct molecular profiles according to IMDC risk stratification.

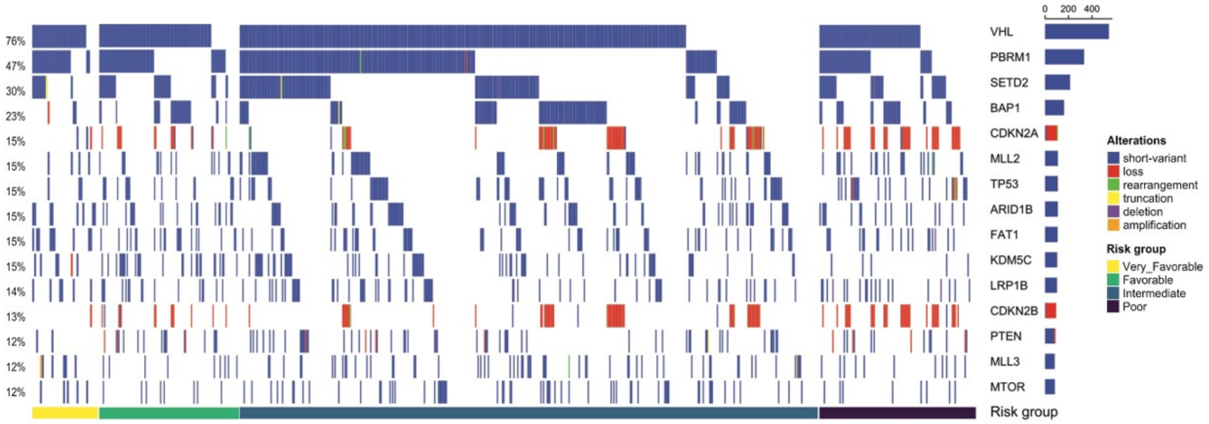

The CoMut plot illustrates distinct patterns of somatic alterations across risk groups, supporting biologically meaningful differences between the very favorable subgroup and higher-risk disease, although these observations are descriptive and hypothesis generating.

© 2026 Zarba M et al. *JAMA Network Open*.

eFigure 2. Distribution of the 7 Molecular Clusters in Each Risk Group and ssGSEA Scores for Angiogenic and Immune Pathways Stratified by IMDC Risk Group

Supplementary fig 2. (A) Distribution of the 7 molecular clusters in each risk group. (B) ssGSEA scores for angiogenic and immune pathways stratified by IMDC risk group. For categorical variables, significance was assessed with Fisher's exact test. For ssGSEA scores, the Wilcoxon rank-sum test was used.

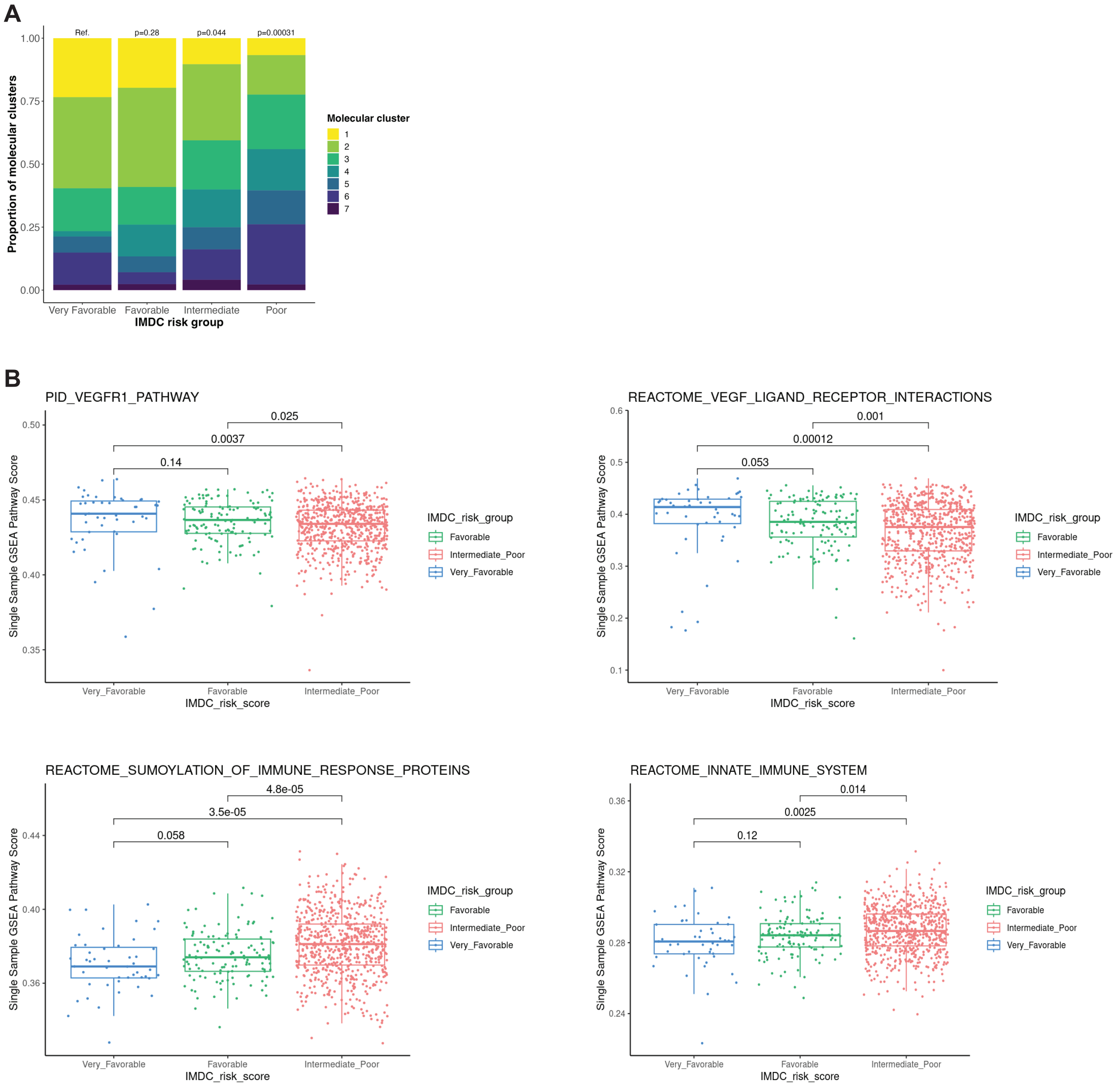

Caption

© 2026 Zarba M et al. *JAMA Network Open*.
