# Supplementary material for: Clinical and Molecular Validation of the Very Favorable IMDC Risk Group in Metastatic Renal Cell Carcinoma
Source: JAMA Netw Open. 2026 Apr 15;9(4):e267030. doi: 10.1001/jamanetworkopen.2026.7030 (PMC13084435; doi:10.1001/jamanetworkopen.2026.7030)
Supplement: Supplement 3. — Data Sharing Statement [file jamanetwopen-e267030-s003.pdf]

Data Sharing Statement

Zarba. Clinical and Molecular Validation of the Very Favorable IMDC Risk Group in Metastatic Renal Cell Carcinoma. *JAMA Netw Open*. Published April 15, 2026.
doi:10.1001/jamanetworkopen.2026.7030

Data
Data available: No

Additional Information
Explanation for why data not available: Data used for this study is propriety of the IMDC and Genentech and it's not going to be released unless previously approved by both groups.
